# A mutation-specific, single-arm, phase 2 study of dovitinib in patients with advanced malignancies

**DOI:** 10.18632/oncotarget.27530

**Published:** 2020-04-07

**Authors:** Matthew H. Taylor, Ajjai S. Alva, Timothy Larson, Sebastian Szpakowski, Das Purkaystha, Alpesh Amin, Linda Karpiak, Sarina A. Piha-Paul

**Affiliations:** ^1^Division of Hematology and Medical Oncology, Knight Cancer Institute, Oregon Health & Science University, Portland, OR, USA; ^2^Division of Hematology and Oncology, University of Michigan, Ann Arbor, MI, USA; ^3^USOR – Minnesota Oncology, Minneapolis, MN, USA; ^4^Novartis Institutes for Biomedical Research, Cambridge, MA, USA; ^5^Novartis Pharmaceuticals Corporation, East Hanover, NJ, USA; ^6^Department of Investigational Cancer Therapeutics, The University of Texas MD Anderson Cancer Center, Houston, TX, USA

**Keywords:** advanced malignancies, basket trial, dovitinib, histology-agnostic, mutation-specific

## Abstract

Background: Receptor tyrosine kinases (RTKs) play key roles in tumorigenesis. The multi-RTK inhibitor dovitinib has demonstrated promising antitumor activity in multiple cancers.

Patients and Methods: In this phase 2, open-label, single-arm study, patients with advanced malignancies with RTK-pathway genetic aberrations whose disease progressed on/following standard treatment received dovitinib (500 mg/day; 5-days-on/2-days-off). The primary endpoint was clinical benefit rate (CBR; complete response, partial response [PR], or stable disease [SD] for ≥ 16 weeks).

Results: Of 80 patients enrolled, common tumors included gastrointestinal stromal tumors (GIST; 20.0%), colorectal cancer (CRC; 18.8%), and ovarian cancer (10.0%). Patients were heavily pretreated (median prior lines = 4; 67.5% had ≥ 3 prior lines). Genetic aberrations included *cKIT* (28.8%), *FGFR3* (15.0%), and *RET* (15.0%). The CBR was 13.8%; one PR (GIST) and 10 SD (adenoid cystic [*n* = 3]; ovarian [*n* = 3]; GIST [*n* = 2]; CRC [*n* = 1]; gastroesophageal junction [*n* = 1]). The most common treatment-related adverse events were fatigue, diarrhea, nausea, and vomiting.

Conclusions: In this heterogeneous patient population, the safety profile was acceptable for dovitinib therapy. A subset of patients with RTK pathway-activated tumors experienced clinical benefit. However, the primary endpoint was not met, suggesting further refinement of predictive biomarkers is required.

## INTRODUCTION

Receptor tyrosine kinases (RTKs), including vascular endothelial growth factor receptor (VEGFR), fibroblast growth factor receptor (FGFR), platelet-derived growth factor receptor (PDGFR), and the proto-oncogene cKIT play multiple roles in tumor growth, development, and survival [1]. Mutations in RTKs that result in aberrant signaling have been linked to tumorigenesis [2]. The prevalence and role of RTK mutations in human cancers has been the subject of a number of clinical studies. Activating mutations in *cKIT* and *PDGFRα* are common in gastrointestinal stromal tumors and are thought to play crucial roles in tumor initiation [3, 4]. Aberrations in FGFRs have been observed across multiple tumor types, most commonly in urothelial, breast, endometrial, squamous lung cancer, and ovarian cancers [1, 5].

Dovitinib (TKI258) is a small molecule multi-kinase inhibitor that targets a number of RTKs, including FGFR, VEGFR, PDGFR, colony stimulating factor 1 receptor (CSF-1R), cKIT, RET, tropomyosin receptor kinase A (TrkA), and fms-related tyrosine kinase 3 (FLT3) [6]. This broad range of targets is the mechanistic basis for the antiproliferative and antiangiogenic effects of dovitinib observed in preclinical studies [6–8]. In the clinic, dovitinib has demonstrated promising activity in a number of cancers, including as a single agent in gastrointestinal stromal tumors [9] and in combination with fulvestrant in advanced breast cancer [10].

In this trial (NCT01831726), dovitinib was investigated as part of the Novartis Signature Program (8 phase 2, signal-finding, basket trials of different single-agent targeted therapies in a genetic alteration-specific manner) [11]. The purpose of this tumor histology-agnostic study was to determine whether dovitinib treatment demonstrated sufficient efficacy in select RTK pathway-activated cancers to support additional studies.

## RESULTS

### Patients

Between August 15, 2013 and December 17, 2015, 80 patients with advanced malignancies were enrolled and received at least one dose of dovitinib. Baseline patient characteristics are shown in Table 1. The median age was 60 years (range: 29–80) and the patient population was heavily pretreated with a median number of 4 prior lines of anticancer therapy (range: 0–14); 67.5% of patients had received at least 3 prior lines. Gastrointestinal stromal tumor (20.0%), colorectal cancer (18.8%), and ovarian cancer (10.0%) were the most common solid malignancies; no patients with hematologic malignancies were enrolled. Tumor type cohorts of at least 4 patients were formed in the following malignancies: gastrointestinal stromal tumor (*n* = 16), colorectal cancer (*n* = 15), ovarian cancer (*n* = 8), adenoid cystic carcinoma (*n* = 7), head and neck squamous cell carcinoma (*n* = 5), non-small cell lung cancer adenocarcinoma (*n* = 4), and thymus cancer (*n* = 4). The most common (in ≥ 15.0% of patients) genetic aberrations that were required for patient enrollment were *cKIT* (28.8%), *FGFR3* (15.0%), and *RET* (15.0%).

**Table 1:** Patient baseline characteristics

Characteristic	Patients (*N* = 80)
**Median age, years (range)**	60 (29–80)
**Sex, *n* (%)**	
Female	40 (50.0)
Male	40 (50.0)
**Race, *n* (%)**	
Caucasian	69 (86.3)
Black	7 (8.8)
Asian	2 (2.5)
Other	2 (2.5)
**ECOG PS, *n* (%)**	
0	38 (47.5)
1	42 (52.5)
**Primary tumor type, *n* (%)**	
Gastrointestinal stromal tumor	16 (20.0)
Colorectal cancer	15 (18.8)
Ovarian	8 (10.0)
ACC	7 (8.8)
HNSCC	5 (6.3)
NSCLC adenocarcinoma	4 (5.0)
Thymus	4 (5.0)
Other/unknown^a^	21 (26.3)
**Prior lines of antineoplastic medication, *n* (%)**	
0	4 (5.0)
1	11 (13.8)
2	11 (13.8)
3	13 (16.3)
4	15 (18.8)
≥ 5	26 (32.5)

^a^Other tumors in fewer than 4 patients were: sarcoma (*n* = 3), central nervous system, lung non-small cell non-adenocarcinoma, neuroendocrine, pancreas, and small intestine (*n* = 2 each), and cervix, gall bladder, gall bladder ducts, gastroesophageal junction, and melanoma (*n* = 1 each). Primary tumor type was unknown in 3 patients. Abbreviations: ACC, adenoid cystic carcinoma; ECOG PS, Eastern Cooperative Oncology Group performance status; HNSCC, head and neck squamous cell carcinoma; NSCLC, non-small cell lung cancer.

At final data cut-off (February 10, 2016) all patients had discontinued treatment, most commonly due to disease progression (61.3%; Supplementary Figure 1). The median duration of dovitinib treatment was 1.78 months (range: 0.1–16.7) with the majority of patients treated for a maximum of 2 months (61.3%). The median duration of follow-up was 29.9 months. Dovitinib dose was reduced in 39 patients (48.8%) with adverse events the most common reason for dose reduction in 37 patients (46.3%). The median relative dose intensity for dovitinib was 100% (range: 40–100).

### Safety

Of all patients who received at least one dose of dovitinib and had at least one post-baseline safety assessment, 74 patients (92.5%) experienced a treatment-emergent adverse event, suspected to be related to study drug (Table 2). The most common treatment-emergent–related adverse events of any grade in ≥ 30% of patients were fatigue (60.0%) and the gastrointestinal disorders diarrhea (55.0%), nausea (53.8%), and vomiting (41.3%). Grade 3/4 treatment-emergent suspected-related adverse events were experienced by 45 patients (56.3%); fatigue was the most common in 14 patients (17.5%). Decreased platelet count, hypertriglyceridemia, and increased gamma-glutamyltransferase were the only Grade 4 adverse events reported in more than 1 patient (2 patients [2.5%] each).

**Table 2:** Treatment-emergent adverse events suspected to be related to dovitinib in ≥ 10% of patients

Adverse event, *n* (%)	Patients (*N* = 80)			
	Grade 1	Grade 2	Grade 3/4	Any grade
**Any adverse event**	5 (6.3)	24 (30.0)	45 (56.3)	74 (92.5)
Fatigue	12 (15.0)	22 (27.5)	14 (17.5)	48 (60.0)
Diarrhea	24 (30.0)	14 (17.5)	6 (7.5)	44 (55.0)
Nausea	22 (27.5)	16 (20.0)	5 (6.3)	43 (53.8)
Vomiting	21 (26.3)	8 (10.0)	4 (5.0)	33 (41.3)
Hypertriglyceridemia	8 (10.0)	9 (11.3)	6 (7.5)	23 (28.8)
Decreased appetite	11 (13.8)	9 (11.3)	2 (2.5)	22 (27.5)
Increased blood AP	5 (6.3)	8 (10.0)	5 (6.3)	18 (22.5)
Increased AST	11 (13.8)	3 (3.8)	1 (1.3)	15 (18.8)
Asthenia	4 (5.0)	6 (7.5)	3 (3.8)	13 (16.3)
Dehydration	2 (2.5)	9 (11.3)	2 (2.5)	13 (16.3)
Decreased weight	6 (7.5)	6 (7.5)	0	12 (15.0)
Increased ALT	9 (11.3)	3 (3.8)	0	12 (15.0)
Increased GGT	3 (3.8)	4 (5.0)	5 (6.3)	12 (15.0)
Hypertension	4 (5.0)	2 (2.5)	4 (5.0)	10 (12.5)
Rash	7 (8.8)	3 (3.8)	0	10 (12.5)
Thrombocytopenia	5 (6.3)	0	4 (5.0)	9 (11.3)
Dysgeusia	6 (7.5)	2 (2.5)	0	8 (10.0)
Increased amylase	5 (6.3)	2 (2.5)	1 (1.3)	8 (10.0)
Increased lipase	5 (6.3)	0	3 (3.8)	8 (10.0)

Abbreviations: ALT, alanine aminotransferase; AP, alkaline phosphatase; AST, aspartate aminotransferase; GGT, gamma-glutamyltransferase.

Treatment-emergent adverse events requiring dose adjustment or interruption, suspected to be related to study drug, were reported in 45 patients (56.3%); these were most commonly (in ≥ 5.0% of patients), fatigue (21.3%), gastrointestinal disorders including nausea (12.5%), diarrhea (11.3%) and vomiting (7.5%), and hypertension (5.0%). Adverse events were the cause of treatment discontinuation in 16 patients (20.0%). Nine patients (11.3%) died during the study or within 30 days of treatment discontinuation; of these, death was the cause of study discontinuation in seven patients (8.8%; Supplementary Figure 1). Patient deaths were due to disease progression (*n* = 5 [6.25%]) and serious adverse events (*n* = 4 [5.0%]). The serious adverse events leading to death were acute thrombotic cerebrovascular event, pulmonary embolism, acute respiratory failure, and cardiac arrest. The cases of acute thrombotic cerebrovascular event and pulmonary embolism were suspected to be related to study drug.

### Efficacy

Clinical benefit was experienced by 11 patients (13.8%); 10 patients (12.5%) had stable disease for at least 16 weeks and 1 patient (1.3%) had a partial response lasting 116 days (Table 3). Stable disease was experienced by patients in the following tumor cohorts: adenoid cystic tumor (*n* = 3), ovarian tumor (*n* = 3), gastrointestinal stromal tumor (*n* = 2), and colorectal tumor (*n* = 1). Outside of the tumor cohorts, stable disease was also reported in an additional patient with a gastroesophageal junction tumor. The following tumor mutations were reported in patients with clinical benefit: *cKIT* (*n* = 1), *CSF-1R* (*n* = 1), *PDGFRα* (*n* = 2), *PDGFRβ* (*n* = 1), *VEGFR1* (*n* = 1), *VEGFR2* (*n* = 2), *FLT3* (*n* = 1), *FGFR2* (*n* = 2), *RET* (*n* = 1), and *TrkA* (*n* = 1).

**Table 3:** Summary of clinical benefit

	Patients (*N* = 80)
Tumor response, *n* (%)	
Complete response	0
Partial response	1 (1.3)
Stable disease (≥ 16 weeks)	10 (12.5)
Progressive disease	47 (58.8)
Non-evaluable	22 (27.5)
**Clinical benefit rate, *n* (%) [95% CI]**^**a**^	11 (13.8) [7.1–23.3]
**Overall response rate, *n* (%) [95% CI]**^**b**^	1 (1.3) [0.0–6.8]

^a^Clinical benefit rate = complete response + partial response + stable disease for at least 16 weeks.

^b^Overall response rate = complete response + partial response. Abbreviation: CI, confidence interval.

The patient with a partial response was a 49-year-old male with a gastrointestinal stromal tumor and metastatic sites in the lung, liver, and jejunum. The best percentage change from baseline in the size of target lesion was –38.6%. He had received 3 prior lines of therapy. Sequencing of a stomach tumor biopsy taken 46.1 months prior to study start per local analysis revealed presence of a *cKIT* mutation; *posthoc* central sequencing analysis highlighted an additional mutation in *VEGFR2*. The patient had received the following therapies between the date of biopsy and first dose of dovitinib: imatinib, sunitinib, and regorafenib.

**Figure 1:**
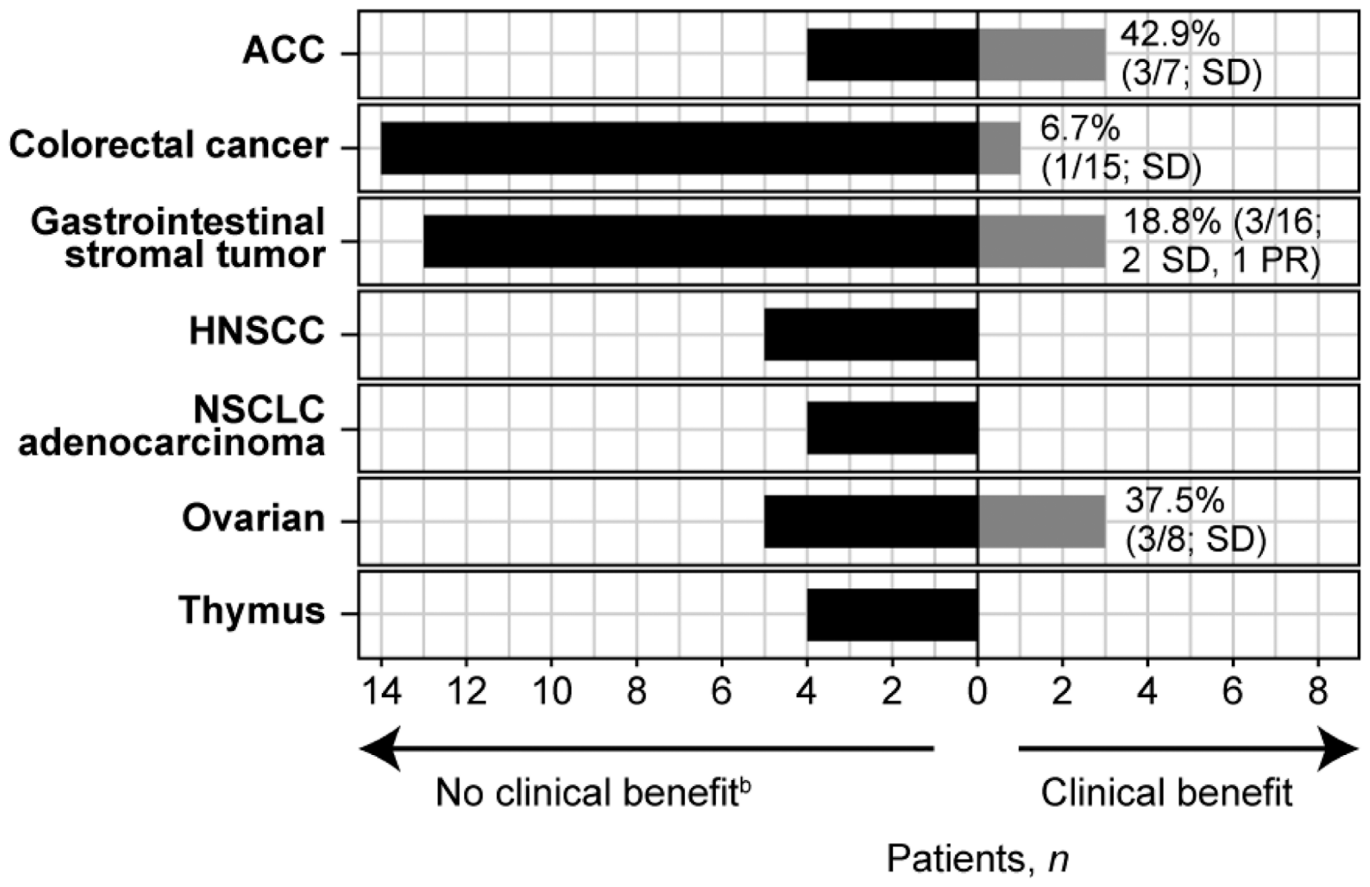
Clinical benefit per tumor type cohort^a^. ^a^Only tumor types with at least 4 patients are included. ^b^Patients for whom clinical benefit was non-evaluable are included under “no clinical benefit” as follows: ACC *n* = 1; Colorectal cancer *n* = 2; Gastrointestinal stromal tumor *n* = 3; HNSCC *n* = 3; NSCLC adenocarcinoma *n* = 1; ovarian *n* = 3; thymus *n* = 3. Abbreviations: ACC, adenoid cystic carcinoma; HNSCC, head and neck squamous cell carcinoma; NSCLC, non-small cell lung cancer; PR, partial response; SD, stable disease.

Median progression-free survival in all patients treated with dovitinib was 2.4 months (95% confidence interval [CI]: 1.8–3.7) with 60 (75.0%) events. The median overall survival was 13.5 months (95% CI: 5.9–14.7) with 34 (42.5%) events. Of the 7 tumor cohorts that enrolled at least 4 patients (adenoid cystic, colorectal, gastrointestinal stromal, head and neck squamous cell carcinoma, lung non-small cell adenocarcinoma, ovarian, and thymus), none passed the prespecified threshold of benefit according to Bayesian analysis and futility was declared (Figure 1).

### Genomic profiling

**Figure 2:**
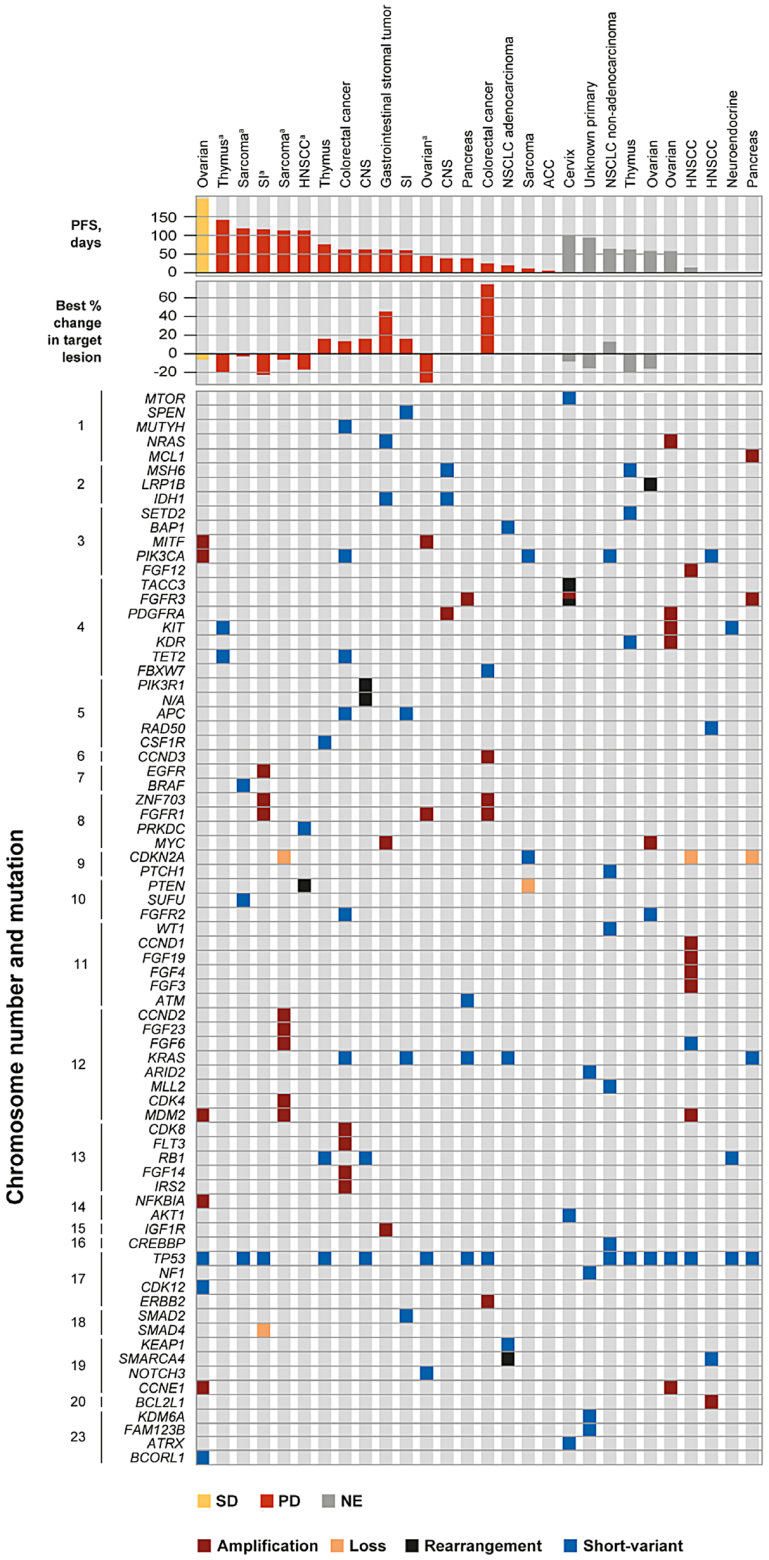
Genomic profile and tumor response in patients with biopsy taken within 12 months of study start. Specific nucleotide substitutions of *PIK3CA*, *PIK3R1*, *KRAS*, and *TP53* are shown in Supplementary Table 2. ^a^Response defined as progressive disease despite decreased size of target lesion due to progression of existing non-target lesions, the appearance of new lesions, or progressive disease in target lesion at end of treatment. In the patients with sarcoma and HNSCC, progressive disease was given as the reason for discontinuation without imaging evidence. Abbreviations: ACC, adenoid cystic carcinoma; CNS, central nervous system; HNSCC, head and neck squamous cell carcinoma; NE, non-evaluable; NSCLC, non-small cell lung cancer; PD, progressive disease; PFS, progression-free survival; SD, stable disease; SI, small intestine.

The most common protocol-defined genetic aberration was in *cKIT* (28.8%; Table 4). Alterations in the following genes were also common (in ≥ 10% of patients): *FGFR3* and *RET* (in 15.0% of patients each), and *FGFR1* and *VEGFR2* (in 10.0% of patients each). The median time from the date of tumor biopsy used for sequencing to first dose of dovitinib was 18.8 months (range: <1–120). The tumor biopsy was obtained within 12 months prior to dovitinib treatment in 28 patients – genomic alterations are shown in Figure 2 with specific nucleotide substitutions detailed in Supplementary Table 2. Of these patients, 13 had received no anticancer therapy from the time of tumor biopsy to the start of dovitinib therapy. The remaining 15 patients had received at least one line of therapy after collection of the biopsy sample; 5 of these biopsy samples were collected at disease onset.

**Table 4:** Protocol-defined genetic aberrations based on local molecular profiling

Genetic aberration, *n* (%)	Patients^a^ (*N* = 80)
*cKIT*	23 (28.8)
*FGFR3*	12 (15.0)
*RET*	12 (15.0)
*FGFR1*	8 (10.0)
*VEGFR2*	8 (10.0)
*FGFR2*	7 (8.8)
*FLT3*	7 (8.8)
*PDGFRα*	7 (8.8)
*PDGFRβ*	5 (6.3)
*TrkA* (*NTRK1*)	5 (6.3)
*CSF-1R*	2 (2.5)
*VEGFR1*	0

^a^Patients may have alterations in more than one gene at baseline; as such, the total may exceed 100%.

Response rates in those patients with recent biopsies (within 12 months of study treatment) included 1 patient who achieved stable disease, 17 patients with progressive disease, and 10 patients who were not evaluable for clinical benefit. The patient with stable disease and ovarian cancer was a 52-year-old female who had previously been treated with 6 lines of therapy including carboplatin, paclitaxel, cisplatin, carboplatin plus gemcitabine, insulin-like growth factor binding protein 2-targeted therapy, cyclophosphamide, and letrozole. The best percentage change from baseline was –5.1%. Local sequencing analysis of a biopsy from the primary ovarian tumor collected 2.5 months prior to the study start, approximately 3 weeks before cessation of letrozole therapy, revealed the presence of *RET* and *TrkA* (*NTRK1*) protocol-defined mutations. The following aberrations in genes known to be implicated in tumor development were also present: *CCNE1*, *MDM2, MITF*, *NFKBIA*, *PIK3CA* amplifications, and short-variant mutations of *BCORL1*, *CDK12*, and *TP53*.

## DISCUSSION

This signal-seeking study assessed the efficacy of dovitinib in patients with solid tumors possessing genetic aberrations in RTKs. The primary endpoint of the clinical benefit rate was not met, with an observed clinical benefit rate of 13.8% including 1 patient who experienced a partial response. Clinical benefit was assessed and compared to historical rates in tumor cohorts of gastrointestinal stromal tumor, colorectal cancer, ovarian cancer, adenoid cystic carcinoma, head and neck squamous cell carcinoma, non-small cell lung cancer adenocarcinoma, and thymus cancer. Although no tumor histology cohort met the prespecified threshold associated with success, it should be noted that the partial response was observed in the presence of *cKIT* and *VEGFR2* mutations. Several patients experienced clinical benefit with tumor mutations in *cKIT*, *CSF-1R*, *PDGFRα*, *PDGFRβ*, *VEGFR1*, *VEGFR2*, *FLT3*, *FGFR2*, *RET*, and *TrkA*.

Overall, the observed safety profile in this heterogenous patient population was acceptable for dovitinib treatment; the reported adverse events are consistent with the known safety profile of dovitinib monotherapy [17, 18]. No new safety signals emerged during this study. Adverse events thought to be related to dovitinib therapy were experienced by 92.5% of patients, the majority were Grade 2 or 3 in severity. Fatigue and gastrointestinal disorders including diarrhea, nausea, vomiting, and decreased appetite were among the most commonly reported adverse events.

With advances in genomic technologies and a growing knowledge of actionable mutations in targeted therapies, the promise of personalized medicine is growing [19]. In this study, an association between tumor genomic alteration and response was precluded by the small sample sizes for each genomic alteration. Additionally, genetic alterations in other signaling pathways may impact dovitinib activity. Patient-derived xenograft models have demonstrated the association between FGF3 and FGF19 upregulation and response to dovitinib [20]. The presence of additional mutations in related signaling pathways may not be reflected in the dataset in this study due to the length of time between tumor biopsy and dovitinib treatment. There were few patients in which tumor biopsies were collected within 12 months of study start and only 13 patients who had received no other therapy between tumor biopsy and dovitinib treatment. In case of the emergence of treatment-related genetic alterations or suppression, real-time/fresh tumor biopsies in future trials would ensure the identified molecular aberrations accurately represent the tumor genomic background at study start.

Overall, dovitinib therapy was well tolerated in this heavily pretreated patient population and clinical benefit was observed in a subset of patients following dovitinib treatment. Future studies may provide additional insights into the role of the tumor genomic background in response to dovitinib therapy.

## MATERIALS AND METHODS

### Study design, treatment, and objectives

This was a phase 2, open-label, basket study in patients with solid or hematologic malignancies with mutations, translocations, or activations in specified RTK pathways whose disease had progressed on or following standard treatment (NCT01831726). Patients were treated with 500 mg/day dovitinib on a 5-days-on/2-days-off schedule with 28-day treatment cycles until unacceptable toxicity, disease progression, death, or treatment discontinuation for any other reason. There were no breaks between dosing cycles. Dose interruptions or reductions were permitted for patients unable to tolerate the starting dose due to drug-related toxicities; a maximum of 2 dose reductions were permitted to 400 mg and 300 mg, as needed with no dose re-escalation permitted. Radiological tumor assessments were performed at baseline, every 8 weeks (± 4 days) for the first 16 weeks on study, every 16 weeks (± 4 days) thereafter, and at the end of treatment. Safety was monitored by physical examinations, laboratory evaluations, and continuous reporting of adverse events according to the Common Terminology Criteria for Adverse Events v4.03.

The primary endpoint was the clinical benefit rate associated with dovitinib treatment per local investigator assessment. Clinical benefit was defined as the proportion of patients who achieved complete response, partial response, or stable disease for at least 16 weeks. Tumor response was assessed according to Response Evaluation Criteria In Solid Tumors (RECIST) v1.1 for patients with solid tumors; and appropriate hematologic response criteria for patients with hematologic tumors [12]. The key secondary endpoint was the overall response rate, defined as the proportion of patients who achieved complete or partial response. Additional secondary endpoints included progression-free survival, overall survival, duration of response, and safety and tolerability. The association between pathway activation and response to dovitinib was an exploratory endpoint.

### Patient population

Adult patients (≥ 18 years) with advanced (surgically unresectable, recurrent, or metastatic) solid or hematologic malignancies with a pre-identified mutation and/or translocation in one or more of the following genes: *cKIT*, *CSF-1R*, *FGFR1/2/3*, *FLT3*, *PDGFRα/β*, *RET*, *TrkA* (*NTRK1*), and/or *VEGFR1/2* were enrolled. Patients with confirmed amplification of *FGFR1/2/3*, *FLT3*, and/or *cKIT* were also included. Patients were excluded if they had a primary diagnosis of any of the following tumor types: urothelial, hepatocellular carcinoma, endometrial carcinoma, metastatic breast cancer, squamous non-small cell lung cancer, renal cell carcinoma, *FLT3*-mutated acute myeloid leukemia, and multiple myeloma.

Patients had progressive disease or relapse at screening, and had received at least one prior treatment for recurrent, metastatic, or locally advanced disease with no viable standard therapy options expected to result in durable response. Patients with solid tumors or lymphoma must have had measurable disease per RECIST v1.1 or Cheson 2007 [13] criteria, respectively. Patients were excluded if they had received prior treatment with dovitinib or had a known hypersensitivity to dovitinib. All patients had an Eastern Cooperative Oncology Group performance status of 0 or 1 and a life expectancy of at least 16 weeks. Patients with a history of pulmonary embolism, untreated deep venous thrombosis within 6 months prior to study start, impaired cardiac function, or clinically significant cardiac disease were excluded. Patients were also excluded if they had brain metastasis or a history of brain metastasis or leptomeningeal carcinomatosis.

### Genomic profiling

Genomic profiling was not part of the screening process. Gene aberrations in patient tumors resulting in pathway activation were pre-identified in a Clinical Laboratory Improvement Amendments-certified laboratory prior to patient consent and trial enrollment. Archival tumor tissue or fresh tumor biopsy for central molecular testing was required prior to the first dose of dovitinib. *Posthoc* molecular profiling was performed centrally using a next-generation sequencing panel of >280 cancer-associated genes [14].

### Statistical analysis

All efficacy endpoints were assessed in the full analysis set, which includes all patients who received at least one dose of study drug. For the primary endpoint, clinical benefit was evaluated in tumor cohorts containing at least 4 patients with a specific tumor histology using a novel Bayesian adaptive statistical design that allowed the dynamic borrowing of information across groups [15, 16]. In each tumor cohort, clinical benefit was analyzed by comparing the achieved clinical benefit rate with a historical control rate in that tumor type. The assumed control rates are shown in Supplementary Table 1. A 90% probability that the observed response rate exceeded the historical response rate was required for a group to be considered a success.

Safety was assessed in all patients who received at least one dose of study drug and had at least one post-baseline safety assessment.

### Ethical oversight

This study was conducted in accordance with the Declaration of Helsinki and was performed in compliance with Good Clinical Practice guidelines. The protocol and all amendments were reviewed and approved by an Independent Ethics Committee and/or Institutional Review Board at each participating site. All patients provided informed consent prior to study start.

## SUPPLEMENTARY MATERIALS



## Abbreviations

CI: confidence interval
CSF-1R: colony stimulating factor 1 receptor
FGFR: fibroblast growth factor receptor
FLT3: fms-related tyrosine kinase 3
PDGFR: platelet-derived growth factor receptor
RECIST: Response Evaluation Criteria In Solid Tumors
RTK: receptor tyrosine kinase
TrkA: tropomyosin receptor kinase A
VEGFR: vascular endothelial growth factor receptor
