# Supplementary material for: A mutation-specific, single-arm, phase 2 study of dovitinib in patients with advanced malignancies
Source: Oncotarget. 2020 Apr 7;11(14):1235–43. doi: 10.18632/oncotarget.27530 (PMC7147086; doi:10.18632/oncotarget.27530)
Supplement: Supplementary file 1 [file oncotarget-11-1235-s001.pdf]

A mutation-specific, single-arm, phase 2 study of dovitinib in patients with advanced malignancies

SUPPLEMENTARY MATERIALS

Supplementary Table 1: Observed and assumed control clinical benefit rates for each tumor type cohort

Tumor type	Observed rate	Assumed control rate[a]
ACC	0.50	0.64
Colorectal cancer	0.08	0.38
Gastrointestinal stromal tumor	0.29	0.50
HNSCC	0	0.45
NSCLC adenocarcinoma	0	0.45
Ovarian	0.60	0.47
Thymus cancer	0	0.70

[a]The assumed control rates by tumor group were generated following systemic research and findings from congresses and literature, where rates were not directly available, they were estimated based on median progression-free survival findings (assuming exponential progression-free survival). Abbreviations: ACC, adenoid cystic carcinoma; HNSCC, head and neck squamous cell carcinoma; NSCLC, non-small cell lung cancer.

Supplementary Table 2: Short-variant mutations in *PIK3CA*, *KRAS*, *RB1*, and *TP53*

Tumor type	Short-variant mutations			
	PIK3CA	***KRAS***	***RB1***	***TP53***
Ovarian	-	-	-	R110fs*39
Thymus	-	-	-	-
Sarcoma	-	-	-	R213*
SI	-	-	-	R273H
Sarcoma	-	-	-	-
HNSCC	-	-	-	-
Thymus	-	-	S829*	C275S<br>R248G
Colorectal cancer	E545K	G12C	-	-
CNS	-	-	D270fs*2	V216M
Gastrointestinal stromal tumor	-	-	-	-
SI	-	G12A	-	-
Ovarian	-	-	-	F212FS*2
CNS	-	-	-	-
Pancreas	-	G12D	-	R273C
Colorectal cancer	-	-	-	R174_E180>R
NSCLC adenocarcinoma	-	G12C	-	-
Sarcoma	H1047L	-	-	-
ACC	-	-	-	-
Cervix	-	-	-	-
Unknown primary	-	-	-	-
NSCLC non-adenocarcinoma	Q75E	-	-	C275fs*70
Thymus	-	-	-	R280I<br>R174S
Ovarian	-	-	-	Y220C
Ovarian	-	-	-	A138V
HNSCC	-	-	-	H179R
HNSCC	E545K	-	-	-
Neuroendocrine	-	-	Splice	P142fs*28
Pancreas	-	G12V	-	M243_G245del

Abbreviations: ACC, adenoid cystic carcinoma; CNS, central nervous system; HNSCC, head and neck squamous cell carcinoma; NSCLC, non-small cell lung cancer; SI, small intestine.

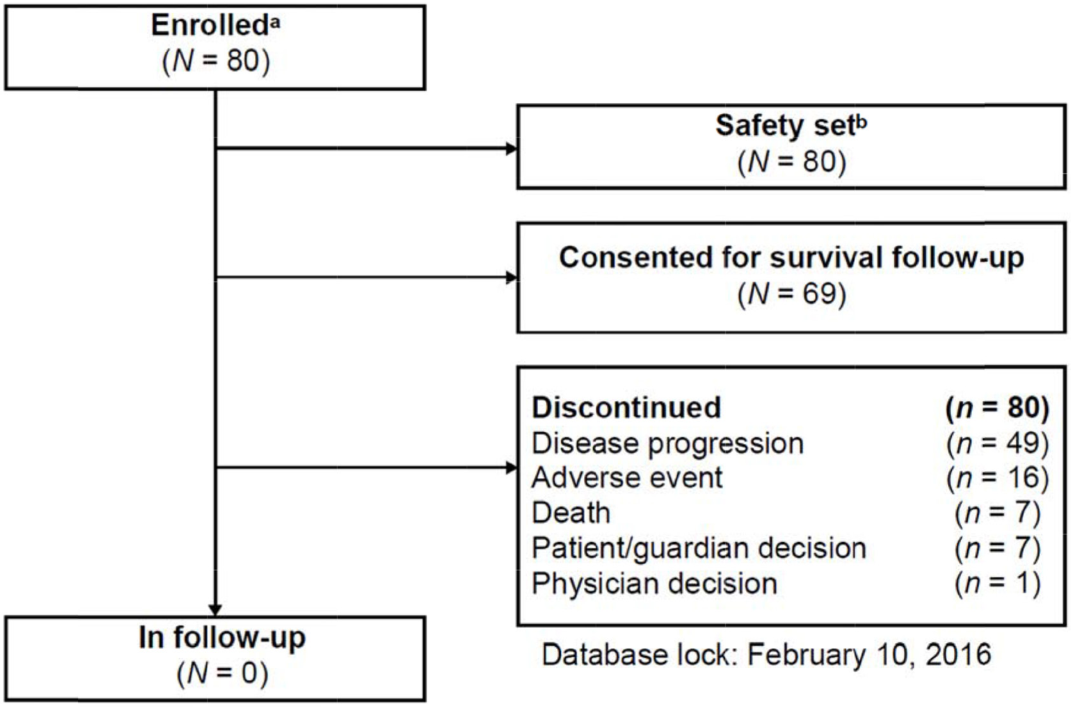

Supplementary Figure 1: CONSORT diagram. [a]Patients who received at least one dose of the study treatment. [b]Patients who received at least one dose of the study treatment and had at least one post-baseline safety assessment.
